# Magnetorheological Elastomer-Based Self-Powered Triboelectric Nanosensor for Monitoring Magnetic Field

**DOI:** 10.3390/nano11112815

**Published:** 2021-10-23

**Authors:** Dong Wan, Ningchen Ma, Taochuang Zhao, Xiaojing Cui, Zhaosu Wang, Hulin Zhang, Kai Zhuo

**Affiliations:** 1 College of Information and Computer, Taiyuan University of Technology, Taiyuan 030024, China; 18771046115@163.com (D.W.); ztttry@163.com (T.Z.); cui85851328@163.com (X.C.); 15841531501@163.com (Z.W.); zhanghulin@tyut.edu.cn (H.Z.); 2 AIEN Institute, Shanghai Ocean University, Shanghai 201306, China; mnc425@126.com

**Keywords:** self-powered, triboelectric, magnetorheological elastomer, magnetic

## Abstract

The adaptable monitoring of the ubiquitous magnetic field is of great importance not only for scientific research but also for industrial production. However, the current detecting techniques are unwieldly and lack essential mobility owing to the complex configuration and indispensability of the power source. Here, we have constructed a self-powered magnetic sensor based on a subtle triboelectric nanogenerator (TENG) that consists of a magnetorheological elastomer (MRE). This magnetic sensor relies on triboelectrification and electrostatic induction to produce electrical signals in response to the MRE’s deformation induced by the variational magnetic field without using any external power sources. The fabricated magnetic sensor shows a fast response of 80ms and a desirable sensitivity of 31.6 mV/mT in a magnetic field range of 35–60 mT as well as preliminary vectorability enabled by the multichannel layout. Our work provides a new route for monitoring dynamic magnetic fields and paves a way for self-powered electric-magnetic coupled applications.

## 1. Introduction

The magnetic field is a vector quantity, which means that it has both magnitude and direction. For many years, researchers have been studying the application of magnetic fields [1,2,3,4,5,6,7,8,9], and have found that they play a significant role in geophysics, space technology, medical applications, and other areas [10,11,12,13]. Currently, there are many methods used to measure magnetic fields, such as magnetic force, hall effect, fluxgate, magnetic resonance, and superconductivity effects. Based on these methods, different principles of using magnetic field measurement instruments have been implemented, and all of these techniques for measuring magnetic fields have their benefits (Table S1) [14]. However, all of the above-mentioned measuring instruments require an external power supply, so it is urgent to develop a self-powered magnetic field monitoring instrument.

MRE is a magnetically sensitive smart composite material prepared from ferromagnetic particles and a polymer-like matrix, whose material properties can be quickly and reversibly controlled by an external magnetic field [15,16,17,18,19,20,21]. Because the matrix of MRE is a solid polymer material, the performance of MRE is simultaneously stable and reproducible. In recent years, a large number of researchers have started to focus on self-powered technologies [22,23,24,25,26]. Among them is TENG: an emerging technology that can directly convert mechanical energy into electrical energy [24,27,28,29,30,31,32]. Since TENG has a high sensitivity to mechanical triggering, many self-powered sensors based on TENG have been designed [16,24,33,34,35,36,37,38]. Here, we designed a self-powered magnetic field monitoring sensor.

In this work, we used styrene ethylene butylene styrene (SEBS) as a substrate and added magnetic fluid to prepare MRE films [39]. In order to measure the strength and direction of the magnetic field, a self-powered sensor based on the MRE of TENG is reported in this paper. The structure and mechanism of the MRE-based TENG are described here in detail, and the material properties of the MRE and the output signals of the sensor are systematically investigated. With the assistance of a programmable platform, the newly designed pointer-based TENG structure enables excellent real-time magnetic field monitoring and unique self-powered capabilities. This work promotes the use of TENG-based sensing applications for magnetic field measurement, which has important implications for IoT, robotics, and AI.

## 2. Discussion and Results

**Figure 1:**
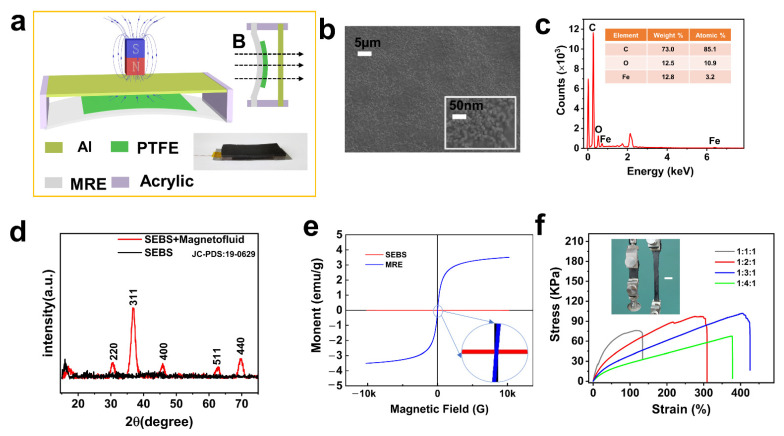
Device models and performance characterization. (**a**) Diagram of device and working model; (**b**) SEM images, (**c**) EDS and (**d**) XRD of the MRE film; (**e**) The hysteresis loop of the SEBS and MRE; (**f**) Stress and strain analysis of the MRE film in different proportions, and photographs of the actual stretching (scale bar is 2 cm).

The structural design of the MRE-based TENG device is illustrated in Figure 1a. When a magnetic field perpendicular to the TENG is applied, the MRE film deforms and drives the PTFE close to the Al under the action of the magnetic field. At the right bottom, a photograph of the fabricated TENG is presented, which shows how the PTFE is conformally pasted on the MRE film. As illustrated in Figure 1b, the scanning electron microscopy (SEM) image of the MRE film shows that the ferruginous particles were uniformly distributed in the SEBS elastomer; this is verified via an EDS test plotted in Figure 1c. The emerging Fe originated from the mixed ferruginous powder, with the observed C and O elements resulting from the SEBS polymer. This is the same as the expected XRD result, indicating that the prepared film consists of Fe_3_O_4_ nanoparticles. As shown in Figure 1d, the diffraction peaks were concentrated at 30.6°, 35.8°, 45.2°, 63.01°, and 70.2°, and are consistent with the face-centered cubic Fe_3_O_4_ (JCPDS No. 19-0629). Figure 1e shows the hysteresis loop of the SEBS and MRE in the range of −10 k to 10 kG, which indicates that the MRE has superparamagnetism and zero coercivity at room temperature; this means that magnetism is completely attributed to the external magnetic field. Moreover, as we expected, SEBS did not change in the presence of the magnetic field. To test the mechanical property, the uni-axial tensile experiments were performed on the prepared MRE films with different component ratios of SEBS powder to liquid paraffin to magnetic fluid as shown in Figure 1f. As the applied strain increases, the stress rises accordingly in a proportional relationship. The stress gradually decreases as the liquid paraffin increases under the same strain condition. The best toughness was achieved at SEBS: liquid paraffin: magnetic fluid = 1:3:1, which could reach 400% strain, and the corresponding stress was 100 KPa. The actual stretching is shown in the illustration of Figure 1f, which indicates that MRE film has excellent tensile properties. In order to deliver a larger stretching range and better toughness, a 1:3:1 blending ratio of SEBS powder, to liquid paraffin to magnetic fluid was chosen for this experiment to make MRE films.

**Figure 2:**
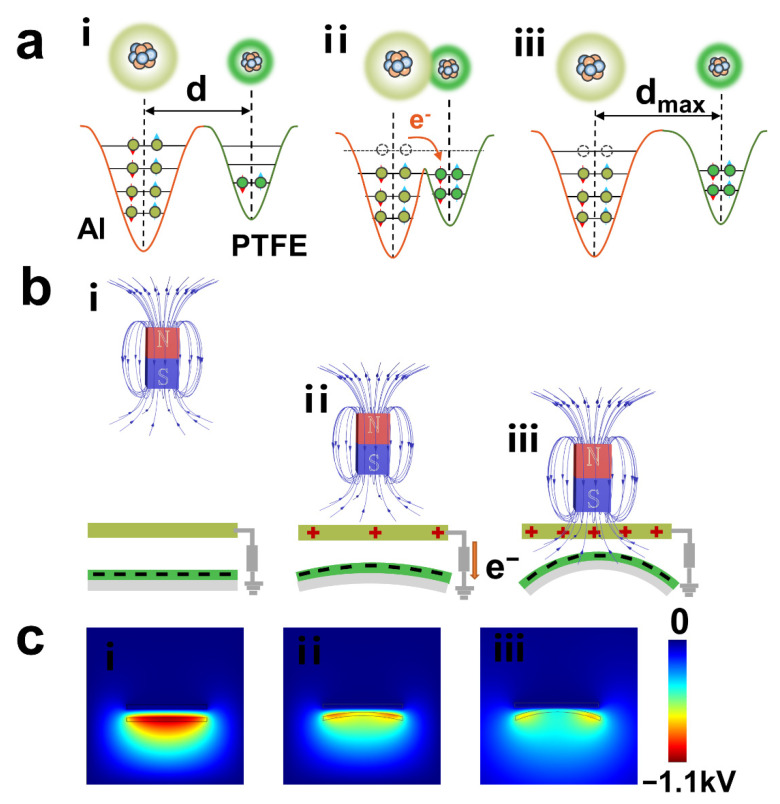
The working mechanism of the triboelectric nanogenerator. (**a**) Schematic of the electron cloud and potential energy profile of two atoms belonging to PTFE and Al, respectively; (**b**) A diagram of the working schematic; (**c**) Simulating the electric potential distribution diagram with COMSOL.

Figure 2a shows the electron cloud and potential energy profile diagram [40]. PTFE is used as a negative triboelectric electric material to build a triboelectric pair with Al, which also works as a single electrode. Until the atoms of PTFE and Al materials come into contact, their electron clouds remain separate (Figure 2a(i)). When PTFE and Al are close together within a magnetic field, the electron clouds of the atoms of the two materials overlap, forming ionic or covalent bonds. Then, electrons can be transferred from one atom to another (Figure 2a(ii)). When PTFE is separated from Al, the transferred electrons are still present on the material surface as electrostatic charges (Figure 2a(iii)). Figure 2b shows the working mechanism of the device. When the magnet is away from the TENG device, the MRE film is not deformed and the gap between the PTFE and Al films is stable. The charges on the surfaces of the PTFE and Al films are balanced, which does not cause a change in the open-circuit voltage (Figure 2b(i)). When the magnet slowly approaches the TENG device, the deformation of the MRE brings the PTFE film closer to the Al film (Figure 2b(ii,iii)). The deformation of the MRE film causes an electrical signal to be outputed by the TENG. The surface charge density between PTFE and Al is set to 1 × 10^−5^ C·m^−2^ by COMSOL software simulation. As illustrated in Figure 2c, when MRE film transitions from the unbending state (i) to the intermediate state (ii) and then to the state with the maximum bending (iii), the potential difference between PTFE and Al interface becomes larger and larger as the separation distance increases, which drives the electrons to flow to the ground. Furthermore, when a gradually increasing magnetic field is applied to the TENG, the maximum force achieved by the mechanical simulation of the MRE is 70 kN/m^2^, as shown in Figure S1.

**Figure 3:**
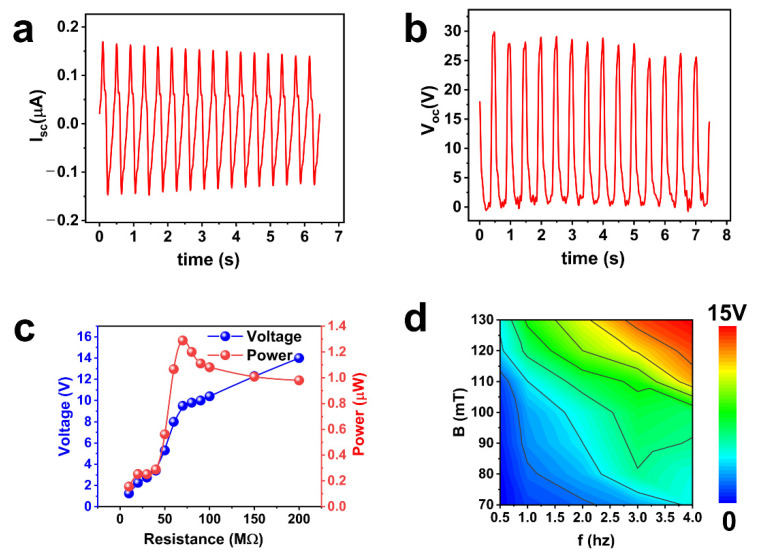
The output performance of TENG. (**a**,**b**) Open-circuit voltage and short-circuit current at the sliding velocity of 16 cm·s^−1^; (**c**) Voltage and power under the different external load resistances; (**d**) The output performance of the TENG under different sliding frequencies and magnetic field strengths.

In order to accurately test the electrical properties of the MRE-based TENG, a programmable linear motor was used. As shown in Figure 3a,b, the open-circuit voltage and short-circuit current are 30 V and 0.18 μA, respectively. In addition, the increase in the load resistance causes the voltage to increase, and the power tends to increase and then decrease, reaching a maximum of 1.3 μW at 80 MΩ, as depicted in Figure 3c. It means that the internal resistance of the TENG is about 80 MΩ, which provides the maximum output power in the external circuit when the load resistance is approximately equal to the internal resistance. Here, we changed the magnetic field strength and the frequency of the magnetic field applied to the TENG to observe the voltage output. As shown in Figure 3d and Figure S2, the higher the magnetic field strength, the higher the voltage output for the same frequency. With the rising frequency, the voltage also increases for the same magnetic field strength.

**Figure 4:**
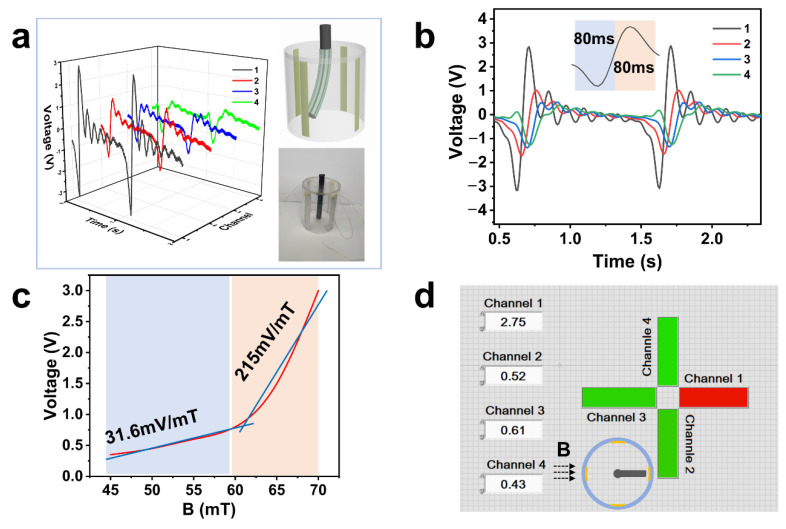
Magnetic field monitoring applications. Measured voltage signals of the sensor when the magnet is close to 1 in 3D (**a**) and 2D (**b**),inset is Sensormodel; (**c**) The output performance of a single channel of the sensor under different magnetic field strengths; (**d**) Mapping image when the magnet is close to channel 1.

After previous tests, we have learned about the output signal characteristics of the MRE-based TENG affected by magnetic fields. Based on the LabVIEW program, a pointer-type magnetic field sensor was designed based on the TENG. The schematic diagram and physical picture are shown in the inset of Figure 4a, which uses an acrylic plate as the base of the cylinder, with Al electrodes symmetrically pasted on all four sides to form 4 channels and numbered. A long cylindrical MRE with multiple PTFE films is fixed in the middle of the cylinder as a pointer. Figure 4a,b plots the voltage output when the magnet is close to channel 1 in 3D and 2D, both of which clearly show a significant rise in voltage peaks and have a fast response of 80 ms. To judge the magnetic field strength by the magnitude of the electrical peak, the output voltage of the device under different magnetic field strengths is illustrated in Figure 4c. The output voltage grows slowly and shows a desirable sensitivity of 31.6 mV/mT when the magnetic field strength is less than 60 mT, while the output voltage increases significantly with a magnetic field strength greater than 60 mT. Durability is another critical factor for the sensor. During 2000 cycles of contact-separation cycles, the voltage reveals a negligible change, as depicted in Figure S3. All of these results suggest that the magnetic sensor is robust enough to work normally under various environmental conditions. To better demonstrate the monitoring capability of the sensor, we performed the test shown in Figure 4d and Figure S4. When a magnetic field is applied in one direction, the corresponding direction channel light turns red, while the other channel’s lights remain green, and the voltage value for each channel is shown in the interface. This result shows that the sensor we designed exhibits excellent real-time magnetic field monitoring and a unique self-powered capability. It also shows that the MRE-based TENG has great potential for magnetic field detection and other smart applications.

## 3. Conclusions

In summary, we invented a self-powered magnetic field monitoring sensor consisting of the MRE-based TENG. MRE film is made by mixing SEBS powder with liquid paraffin and magnetic fluid in a certain mass ratio and then heating at high temperature to make an MRE film. Simulating the magnetic field environment, the MRE-based TENG exhibits good output performance with an open-circuit voltage and short-circuit current of 16 V and 0.18 μA, respectively, and a maximum output power of 1.3 μW at 80 MΩ. Finally, with the assistance of a programmable platform, the pointer-based TENG structure was designed to achieve excellent real-time magnetic field monitoring and a unique self-powered capability. The fabricated magnetic sensor shows a fast response of 80 ms and a desirable sensitivity of 31.6 mV/mT in a magnetic field range of 35–60 mT as well as the preliminary vectorability enabled by the multichannel layout. Our work provides a new route of magnetic field measurements and further pushes the application of triboelectric technology in future sensing.

## 4. Experimental Section

Preparation of MRE film: Firstly, SEBS powder (TSRC Nantong Industrial Co., Ltd., Nantong, China) was mixed with liquid paraffin (Shangqiu Liangfeng Health Care Co., Ltd., Shangqiu, China) and magnetic fluid (Ink king magnetic nanofluid company, Jiaxing, China) in a certain mass ratio and mixed in a beaker to obtain a homogeneous mixture. A certain amount of the mixture was put into a porcelain square with a length, width, and height of 60 mm, 30 mm, and 20 mm, respectively. Then, the porcelain ark was placed in a high-temperature resistance furnace (Shanghai Boxun Industrial Co., Ltd., Shanghai, China) and heated to 225 °C or 30 min. Finally, the porcelain ark was left at room temperature until the molten was cooled, solidified, and peeled from the porcelain ark carrier to obtain the MRE film.

Production of sensor: we used an acrylic cylinder with a diameter of 45 mm and a height of 60 mm as the housing for the magnetic field monitoring sensor. A cylinder MRE with a diameter of 7 mm and a height of 55 mm was fixed in the middle of the cylinder on top of the housing. Four 55 × 10 mm Al sheets, which serve as positive friction material and electrodes, were evenly attached to the inner wall of the housing as four channels. Multiple pieces of 1 mm × 55 mm PTFE films were attached around the cylindrical MRE as the negative friction material.

Output performance measurements: The MRE morphology was characterized by the SEM technique (Hitachi SU8020, Tokyo, Japan). Mechanical analysis was performed using a dynamic mechanical analyzer (Mark-10 Corporation, Copiague, NY, USA) based on an M5-20 dynamometer. In addition, all electrical measurements were performed through a programmable Labview platform consisting of a Keithley 6514, Stanford SR570, and a data acquisition module.

## Supplementary Materials

The following are available online at https://www.mdpi.com/article/10.3390/nano11112815/s1, Figure S1: Simulation drawing of stress and strain experiment, Figure S2: The output performance of the TENG under different sliding frequencies and magnetic field strengths in 2D, Figure S3: The cycle tests of the TENG devices, Figure S4: Mapping image when the magnet is close to channels 2, 3 and 4, Table S1: Comparison of the achievement of our sensor with other competing sensing technologies.
