# Supplementary material for: Magnetorheological Elastomer-Based Self-Powered Triboelectric Nanosensor for Monitoring Magnetic Field
Source: Nanomaterials (Basel). 2021 Oct 23;11(11):2815. doi: 10.3390/nano11112815 (PMC8623981; doi:10.3390/nano11112815)
Supplement: Supplementary file 1 [file nanomaterials-11-02815-s001.zip › nanomaterials-1411613-supplementary.pdf]

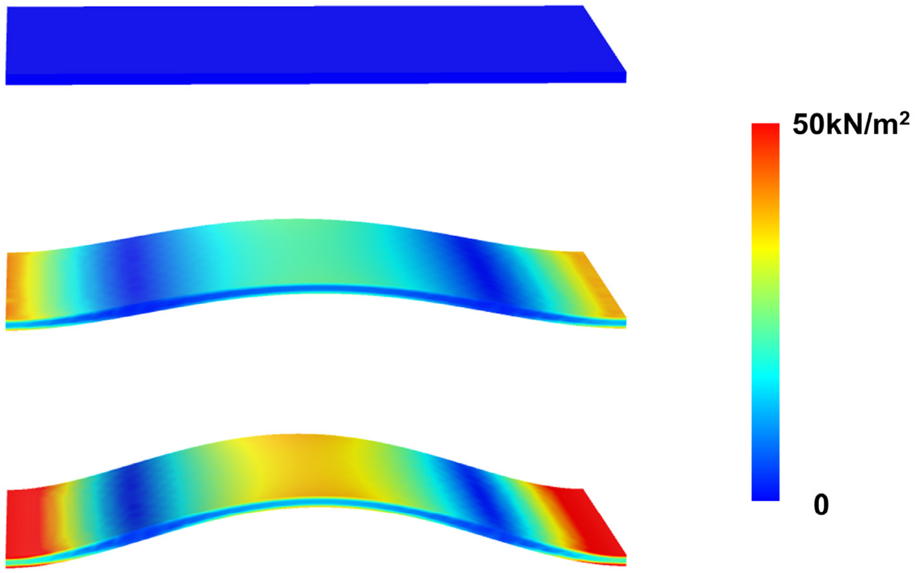

Figure S1. Simulation drawing of stress and strain experiment.

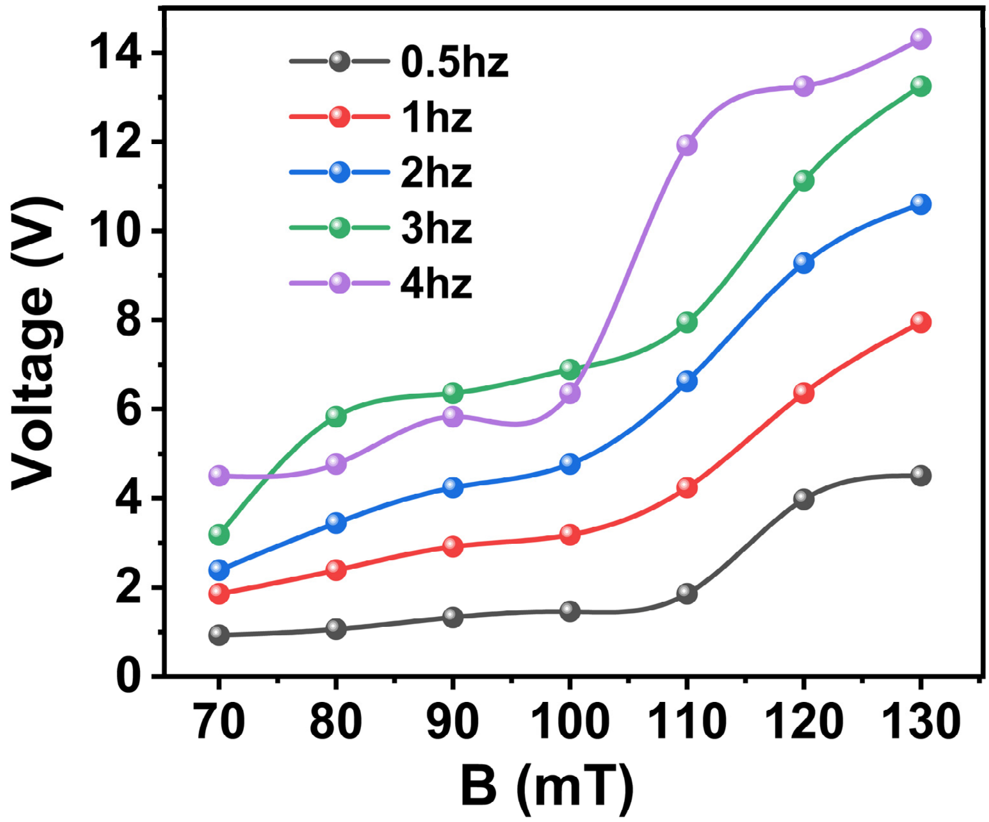

Figure S2. The output performance of the TENG under different sliding frequencies and magnetic field strengths in 2D.

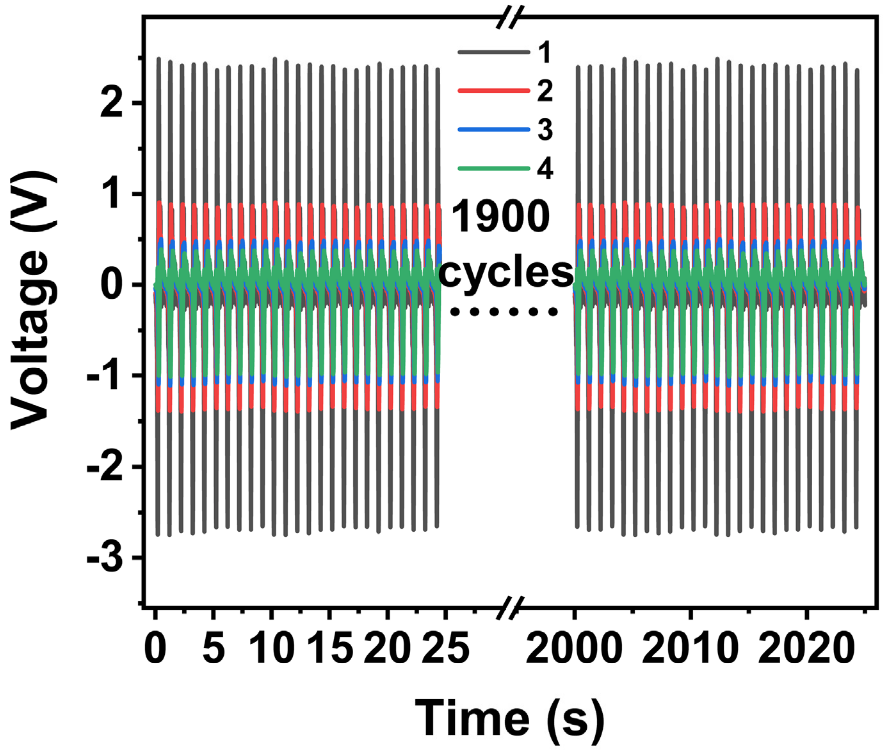

Figure S3. The cycle tests of the TENG devices (the time for each cycle is 1s).

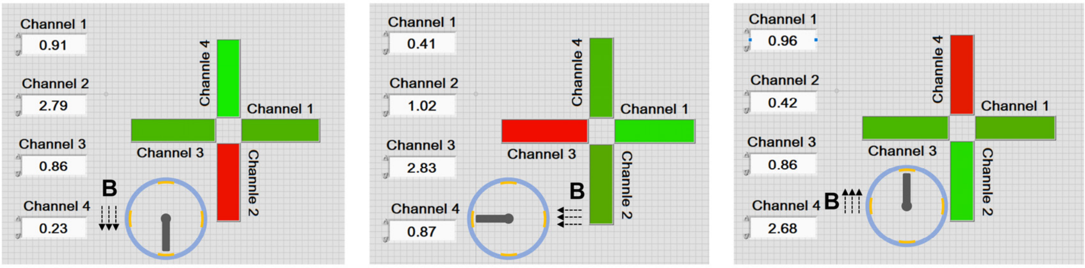

Figure S4. Mapping image when the magnet is close to channels 2, 3 and 4.

Table S1. Comparison of the achievement of our sensor with other competing sensing technologies.

Techology	Representative instrument	Achievement
TENG	Our sensor	Self-powerd
Magnetic force	Torque Magnetometer	Compare and detect magnetic fields
Hall effect	Hall effect magnetometer	Working under various environmental conditions.
Fluxgate	Fluxgate magnetometer	Measurement of weak magnetic field
Magnetic resonance	Nuclear magnetic resonance magnetometer	High accuracy
Superconductivity effects	Superconducting quantum magnetometer	Higher sensitivity
